# Advanced tandem mass spectrometry in metabolomics and lipidomics—methods and applications

**DOI:** 10.1007/s00216-021-03425-1

**Published:** 2021-06-18

**Authors:** Sven Heiles

**Affiliations:** Institute of Inorganic and Analytical Chemistry, Justus Liebig University Giessen, Heinrich Buff Ring 17, 35392 Giessen, Germany

**Keywords:** Tandem mass spectrometry, Lipidomics, Metabolomics, Mass spectrometry imaging, HPLC, Biopolymers/lipids

## Abstract

Metabolomics and lipidomics are new drivers of the omics era as molecular signatures and selected analytes allow phenotypic characterization and serve as biomarkers, respectively. The growing capabilities of untargeted and targeted workflows, which primarily rely on mass spectrometric platforms, enable extensive charting or identification of bioactive metabolites and lipids. Structural annotation of these compounds is key in order to link specific molecular entities to defined biochemical functions or phenotypes. Tandem mass spectrometry (MS), first and foremost collision-induced dissociation (CID), is the method of choice to unveil structural details of metabolites and lipids. But CID fragment ions are often not sufficient to fully characterize analytes. Therefore, recent years have seen a surge in alternative tandem MS methodologies that aim to offer full structural characterization of metabolites and lipids. In this article, principles, capabilities, drawbacks, and first applications of these “advanced tandem mass spectrometry” strategies will be critically reviewed. This includes tandem MS methods that are based on electrons, photons, and ion/molecule, as well as ion/ion reactions, combining tandem MS with concepts from optical spectroscopy and making use of derivatization strategies. In the final sections of this review, the first applications of these methodologies in combination with liquid chromatography or mass spectrometry imaging are highlighted and future perspectives for research in metabolomics and lipidomics are discussed.

**Graphical abstract:**

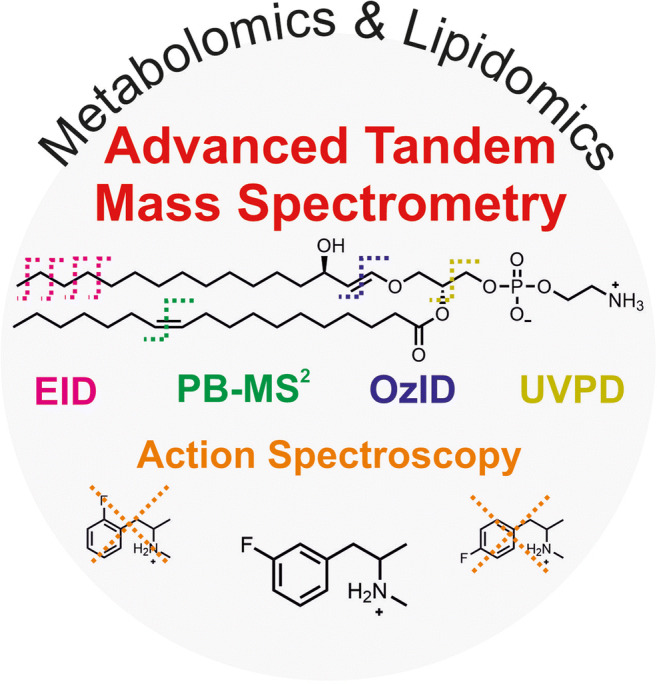

## Introduction

Metabolites are ubiquitous in living organisms, essential for the survival of microorganisms as well as animals and plants. They are used for energy production and storage, act as signaling molecules, serve as cofactors, determine the structural integrity as well as biophysical properties of cell membranes, and can trigger epigenetic regulation processes [1–3]. Metabolites are all substances that are catabolically processed to release energy in order to fuel cellular machineries or are anabolically synthesized compounds to serve specific biochemical functions [4]. Therefore, the metabolome, the entirety of all metabolites, comprises a multitude of endogenous compounds for a given organism but metabolites can also originate from exogenous sources such as microorganisms or xenobiotics [5]. Metabolites can roughly be divided into a water-soluble and water-insoluble fraction. The latter group comprises all lipid entities that define the lipidome [1]. As metabolites are substrates of orchestrated enzymatic cascades and as such are downstream products of biochemical actions, they are lower in molecular weight than nucleic acids and proteins but no less complex to analyze. The complexity arises due to the functional, structural, and chemical diversity of metabolites that often complicates comprehensive analysis of the metabolome. However, technological and methodological improvements witnessed in the last two to three decades have addressed many shortcomings of previous bioanalytic methods establishing metabolomics and lipidomics as new drivers of the omics era.

This is mainly due to the fact that the metabolome and lipidome have been recognized to provide molecular fingerprints for phenotypic characterization. For example, lipid profiles were used by Saudemont et al. to differentiate healthy, necrotic, and cancerous tissue in sarcoma biopsies [6]. These metabolically defined phenotypes can aid real-time diagnosis and help to monitor disease progression, and some of the features that are associated with phenotypes can serve as biomarkers. In a study by Globisch et al., a state-of-the-art metabolomic workflow was used to identify the metabolite N-acetyltyramine-O,β-glucuronide as a characteristic biomarker for the nematode *Onchocerca volvulus*, which is the main cause for the neglected tropical disease river blindness [7]. But beyond phenotyping and biomarker discovery, bioanalytic tools for metabolome and lipidome analysis have made an impact by furthering the mechanistic understanding of the involvement of metabolites in biochemical processes. In a large-scale study by Picotti and co-workers, protein-metabolite interactions and novel metabolite binding modes were identified including the effect of fructose-1,6-bisphosphate-PEP synthetase regulatory protein interactions on the glycolytic flux [8]. These examples demonstrate that metabolome and lipidome analysis can impact numerous fields of research and applications. Recent efforts in the field are directed towards streamlining data acquisition as well as analysis and extend the field of metabolomics and lipidomics beyond global characterization and towards revealing spatial metabolomics and identifying biochemically active metabolites [2, 9]. One of the most prominent tools for global as well as spatial metabolomics is mass spectrometry (MS).

This is because MS combines high measurement speed (typically 5–40 spectra/s) with the sensitivity of ion detection, and the ability to separate as well as identify ionized analytes by mass-to-charge ratios (*m*/*z*). Hyphenated techniques such as liquid chromatography (LC), gas chromatography (GC), and/or ion mobility spectrometry (IMS) can further improve the performance of MS-based metabolomic studies by separating isobars/isomers and reducing matrix effects. Due to the beneficial bioanalytic performance characteristics of MS, recent years have seen a surge in the development of MS-based metabolomic and lipidomic workflows. These developments have been extensively reviewed by experts in the field. Therefore, the author will refer the reader to these reviews where appropriate. Metabolomics methods can roughly be grouped into three categories: (a) direct infusion studies, (b) methods employing chromatographic separation, and (c) mass spectrometry imaging (MSI) investigations. In direct infusion, analytes, often ionized with electrospray ionization (ESI), matrix-assisted laser desorption/ionization (MALDI), or desorption electrospray ionization (DESI), are introduced into mass spectrometers without prior separation to maximize sample throughput. For metabolite separation, LC and GC systems are routinely employed prior to ESI and electron impact ionization (EI)/chemical ionization (CI), respectively. In order to reveal local metabolite or lipid alterations, MSI methods are utilized that can typically be regarded as a form of direct infusion method that offers spatial metabolite distributions.

**Fig. 1:**
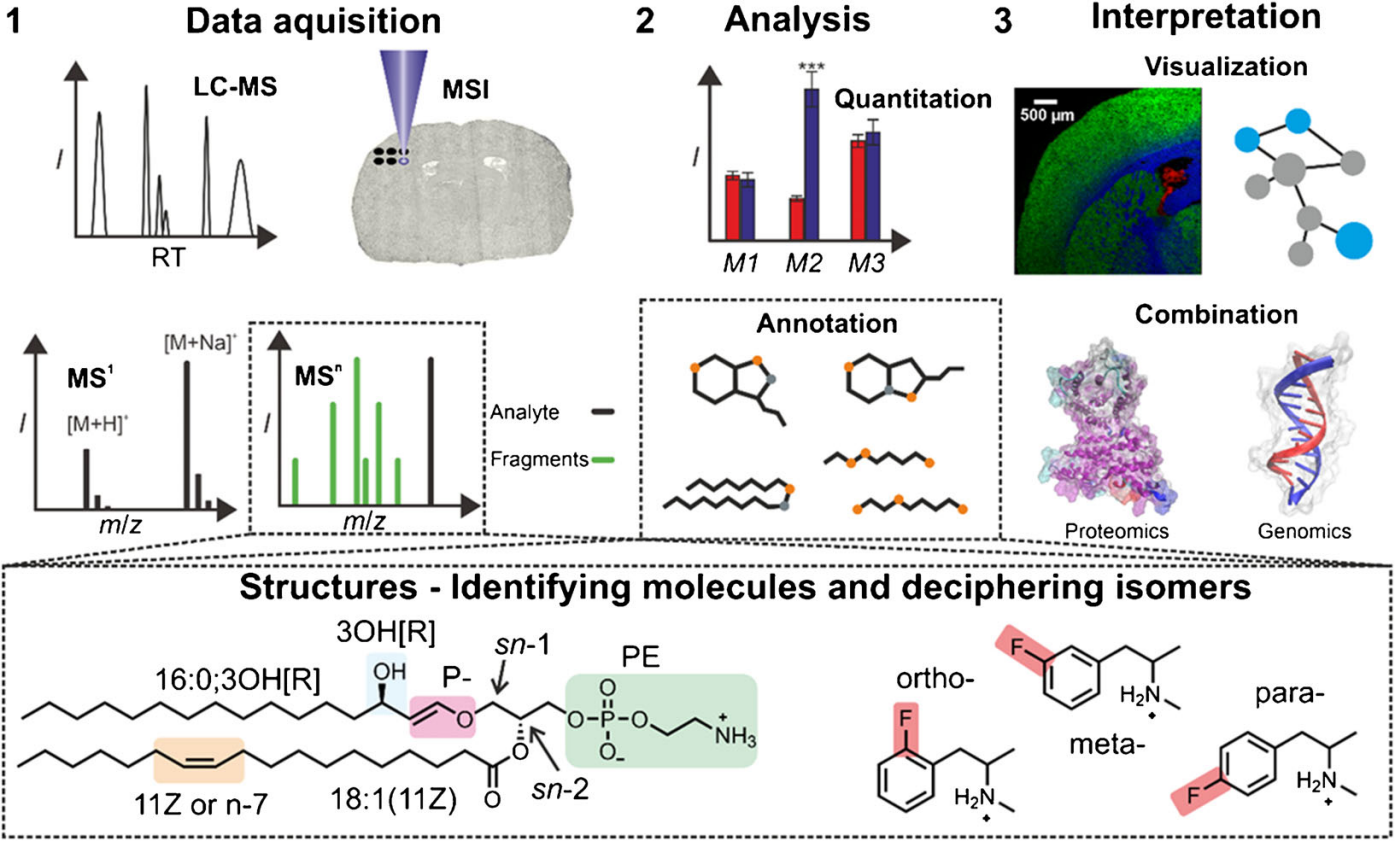
An overview of MS-based metabolomic and lipidomic workflows. (1) Mass spectrometric data is collected after sample preparation. Typically, MS^1^ and MS^n^ datasets are recorded. In case LC-MS or MSI is performed, every mass spectrum is associated with RTs and sampling positions, respectively. (2) Mass spectrometric intensities are used to quantitate fold changes and assign mass spectrometric features to specific compounds or compound groups. (3) In combination with additional data, e.g., from other omics disciplines (images created with VMD [10]) the data is visualized in order to interpret compound distributions or identify alterations of biochemical pathways. Dashed boxes: although MS^n^ results and database searches yield a list of plausible annotations, some structural details are not resolved. Examples are the structures of PE-P 16:0;3OH[R]/18:1(11Z) and fluoromethamphetamine isomers that are not fully resolved based on CID-MS^n^ results

A simplified untargeted metabolomic workflow, i.e., charting of as many metabolites as possible, is shown in the upper half of Fig. 1. For most aspects of sample preparation and the field of GC-MS-based metabolomics/lipidomics, readers are referred to the excellent overview articles by Drouin et al. as well as Beale, Dias, and co-workers, respectively [11, 12]. After the samples have been prepared from body fluids, cells, or tissues, mass spectrometric data is recorded. From these experiments, *m*/*z* values of one or multiple metabolite adducts and corresponding mass spectrometric intensities are obtained (Fig. 1—Data acquisition) and are potentially stored together with retention time (RT) or sampling position for LC-MS or MSI, respectively. The *m*/*z* values and isotopic distribution of metabolites recorded with instruments that offer high mass resolution and mass accuracy allow to assign sum formulae based on accurate mass measurements. In case chromatographic separation is employed, assignments are corroborated by comparison of RTs with those of authentic metabolite standards. Next, mass spectrometric signal intensities are utilized to quantitate metabolite fold changes between samples or sample regions (Fig. 1—Analysis). These untargeted metabolite screens can be repeated in targeted metabolomic experiments for selected analytes. For these targeted procedures, specialized workflows relying on isotope tracing, metabolite derivatization, and biochemical assays have been developed [2, 13]. Beyond this point, data interpretation can strongly differ in metabolomics and often depends on the problem at hand. Some representative examples are shown in Fig. 1—Interpretation. Often metabolomic and lipidomic data is combined with results for the same sample from other omics disciplines, such as genomics and/or proteomics. Data analysis and automated combination with other omics data to facilitate interpretation is currently one of the bottlenecks in metabolomics/lipidomics. Therefore, a major aspect of current metabolomic and lipidomic research is the development of new and more powerful software solutions. Progress in this field has been recently reviewed by Uppal et al., Ren et al., and Alexandrov [9, 14, 15]. However, the discussion so far was restricted to measurements of *m*/*z* values and intensities of intact metabolite ions, the so-called MS^1^ experiments. Metabolomics on the MS^1^ level only reveals sum formulae. Comparison of MS^1^ data to databases enables assignment of features to a limited number of compounds with the correct sum formula. These associations are typically referred to as annotations. To correlate metabolic alterations with biological effects and eventually link metabolites to biochemical functions, structure identification is pivotal. This is particularly important for features that have not been identified before [16], biomarkers or bioactive metabolites. Compared to other metabolomic/lipidomic platforms [17], MS itself only offers limited insight into metabolite structures. To circumvent this shortcoming, tandem MS (MS^n^), first and foremost collision-induced dissociation (CID) [18], is routinely employed. Tandem MS allows to dissociate selected *m*/*z* features by means of gas-phase ion activation. Resulting product ions aid metabolite structure annotations. However, the number and identity of fragment ions and consequently the information about metabolite structures depend on the employed tandem MS method. Most modern mass spectrometers give access to CID methods that induces ion dissociation upon neutral-ion collisions with collision energies of typically (1–100) eV [19]. This mostly results in thermodynamically controlled ion fragmentation not necessarily revealing all structural details of metabolites. This can complicate comprehensive structural characterization or discrimination of metabolites.

This review, thus, aims to critically review the progress, opportunities, applications, and shortcomings in the field described here as “advanced tandem mass spectrometry” in the last 15 years, with special emphasis on the last 5 years. For the author, this comprises all tandem mass spectrometric methods that go beyond metabolite and lipid characterization with vendor-implemented CID units. The goal is not to provide a comprehensive review or describe all developed instruments but rather to provide the reader with concepts and ideas at the heart of selected advanced tandem MS tools. The article will include discussions of tandem MS methods that rely not only on activation of metabolite ions with electrons and photons but also gas-phase ion/ion or ion/molecules reactions, ion spectroscopy, and analyte derivatization prior to ionization. The concepts of these tools will be discussed and benefits and drawbacks will be highlighted by detailing first applications for structure elucidation of lipids, agrochemicals, illicit drugs, and pharmaceuticals. Finally, the current challenges and shortcomings of these methods, with special emphasis on adapting them for routine LC-MS and MSI workflows, will be detailed and potential future research directions are outlined.

## Mass spectrometric interrogation of metabolite and lipid structures

Despite the many benefits of MS in the field of metabolomics and lipidomics such as high sensitivity and throughput, structural characterization of metabolites and lipids is not the strong suite of MS-based methodologies. Although collision-cross sections (CCSs) provided by IMS [20] and spectral similarity measures employed in bioinformatics tools [21] are emerging as valuable additions for structure elucidation, CID-MS^n^ is at the core of most studies that aim to decipher the molecular makeup of metabolites in untargeted investigations. This is because CID activation of metabolite ions can result in structure diagnostic fragment ions with characteristic fragment ion intensities. These mass spectrometric fingerprints, with aid of fragmentation rules or comparison to authentic standard tandem mass spectra, are used to confidently annotate mass spectrometric features to corresponding metabolites or lipids as outlined by Schymanski et al. [22] These quality measures for structure elucidation via MS have proven very powerful over the years but run into problems if specific structural features of metabolites do not fragment during CID or fragment without providing structural information.

This can be showcased for the glycerophospholipid (GP) PE P-16:0;3OH[R]/18:1(11Z) and isomeric drug molecules 2-fluoromethamphetamine, 3-fluoromethamphetamine, and 4-fluoromethamphetamine shown in Fig. 1—dashed box. The nomenclature for these compounds and for all others in this review, is according the framework established by the LIPIDMAPS consortium (except for C=C positions for which the *n-x* nomenclature is sometimes used) [23] and used by the human metabolome database (HMDB) [24]. For the GP shown in Fig. 1—dashed box, CID experiments enable confident assignment of the lipid head group and the FA composition. In contrast, identification of C=C bond (DB) positions as well as geometry, differentiation between vinyl-ether-linked hydroxylated, ether-linked hydroxylated unsaturated or saturated FA moieties, the hydroxylation position as well as stereochemistry, or relative quantification of *sn*-linkage isomers is typically very challenging with CID methods. Even if hyphenated methods are used and authentic standards are available, full characterization of all structural features is not always possible without involving spectroscopic tools that often require purified compounds. If authentic standards are not available, no appropriate database entries exist, or if the sheer number of metabolites in untargeted metabolomic studies makes comparison to standards not feasible, structural characterization by CID-MS^n^ will often not suffice.

These statements are not only true for GPs but also for other lipids or metabolites such as the fluoromethamphetamine isomers that only differ by the fluorine position (Fig. 1—dashed box). Although fluorine position isomers are separated in GC runs, EI-MS spectra of these isomers are virtually indistinguishable [25]. To overcome these issues, researchers have developed bioinformatic tools that use fragmentation rules [26], predict metabolite CID-MS^n^ spectra [27, 28], or use deep neuronal networks to assign mass spectrometric features to metabolite groups or specific compounds [21]. Alternatively, CCS values from IMS experiments can be utilized to add an additional descriptor for structure annotation as reviewed by Harris et al. as well as Yost and co-workers [29, 30]. Another strategy to facilitate compound identification of metabolites and lipids is the development and use of new MS^n^ strategies as detailed in the following. The discussed “advanced tandem MS” strategies are summarized in Table 1.

**Table 1:** Advanced tandem MS tools and corresponding abbreviations for metabolomics and lipidomics discussed in this review. Bold font highlights the methods mainly discussed in the review

Tandem MS classification	Method name or description (method abbreviation)
Electron-based	Electron capture dissociation (ECD); electron impact excitation of ions from organics (EIEIO); **electron-induced dissociation (EID)**
Photon-based	**IR multiple photon dissociation (IRMPD) with fixed wavelength; ultraviolet photodissociation (UVPD) with 157 nm, 193 nm, and 213 nm; radical-directed dissociation (RDD)**
Ion/ion reactions	Electron transfer dissociation (ETD); **charge transfer dissociation (CTD)**; **charge inversion reactions**
Ion/molecule reactions	**Ozone-induced dissociation (OzID)**; radical-induced dissociation; **functional group selective reactions**
Derivatization prior to tandem MS	**Paternò–Büchi (PB) reaction**; epoxidation; ozonolysis; hydroxylation; **Girard’s reagent T derivatization**
MS-based spectroscopy	IRMPD as well as UVPD action spectroscopy at **room temperature** and at cryogenic temperatures

## Electron-based fragmentation

Unlike CID, in which excess energy introduced by collisions between ions and neutral gas is redistributed within the heat bath of activated molecules, electron-based fragmentation tools rely on interactions between analyte ions and electrons. A prominent tandem MS tool in bioanalytic MS that uses electrons to fragment positively charged precursor ions is electron capture dissociation (ECD) pioneered by McLafferty and co-workers [31]. In ECD, one electron is captured by a positively charged ion. In this process, charge state reduction takes place resulting in the formation of a radical. Ion activation is mainly connected to the release of excess energy associated with radical formation and is only minimally affected by the electron kinetic energy as electrons with kinetic energies between 0 and 3 eV are employed. Subsequently, fragments are formed by intramolecular radical rearrangements. These methods and related variants such as hot-ECD [32] are routinely employed in proteomics and glycoproteomic workflows as they often yield complementary fragments to CID. Additionally, many commercial mass spectrometers offer ECD modules. ECD and fragmentation of radical ions have been reviewed in detail by Marshall and co-workers as well as Tureček and Julian [33, 34]. The biggest caveats of these methods for metabolomics and lipidomics are the charge reduction of positively charged ions and the inability to study negative ions. As most metabolites and lipids only form singly charged ions that are neutralized in ECD, alternative electron-based tandem MS strategies are required.

Therefore, multiple groups have explored the impact of varying electron kinetic energy on tandem MS results. For example, Yoo et al. showed that singly charged negative peptide ions can capture one electron prior to dissociation at kinetic energies between 4 and 6 eV [35]. The efficiency of this negative-ion ECD event, however, strongly depends on the identity of the negative ion. At kinetic energies between 10 and 25 eV, multiple competing processes, which depend on the experimental implementation of the method as well as the studied analyte, can yield fragment ions. Collisions between electrons and singly charged ions in this energy regime can result in electron-impact ionization and/or electronic excitation of ions. Back-reflected secondary electrons, excited metastable electronic states, or the excess energy deposited upon electron-ion collision can all result in tandem mass spectra with a large number of fragment ions. Different terms have been established for these electron-based tandem MS methods. Originally introduced as electron impact excitation of ions from organics (EIEIO) [36], the related method electronic excitation dissociation has also been reported [37]. In this review, the author will address all these methods with the term electron-induced dissociation (EID) [38]. As EID methods allow to fragment singly charged precursors and often yield structure diagnostic fragments not observed with CID, EID-MS^n^ can aid metabolite and lipid structure assignments [39, 40].

**Fig. 2:**
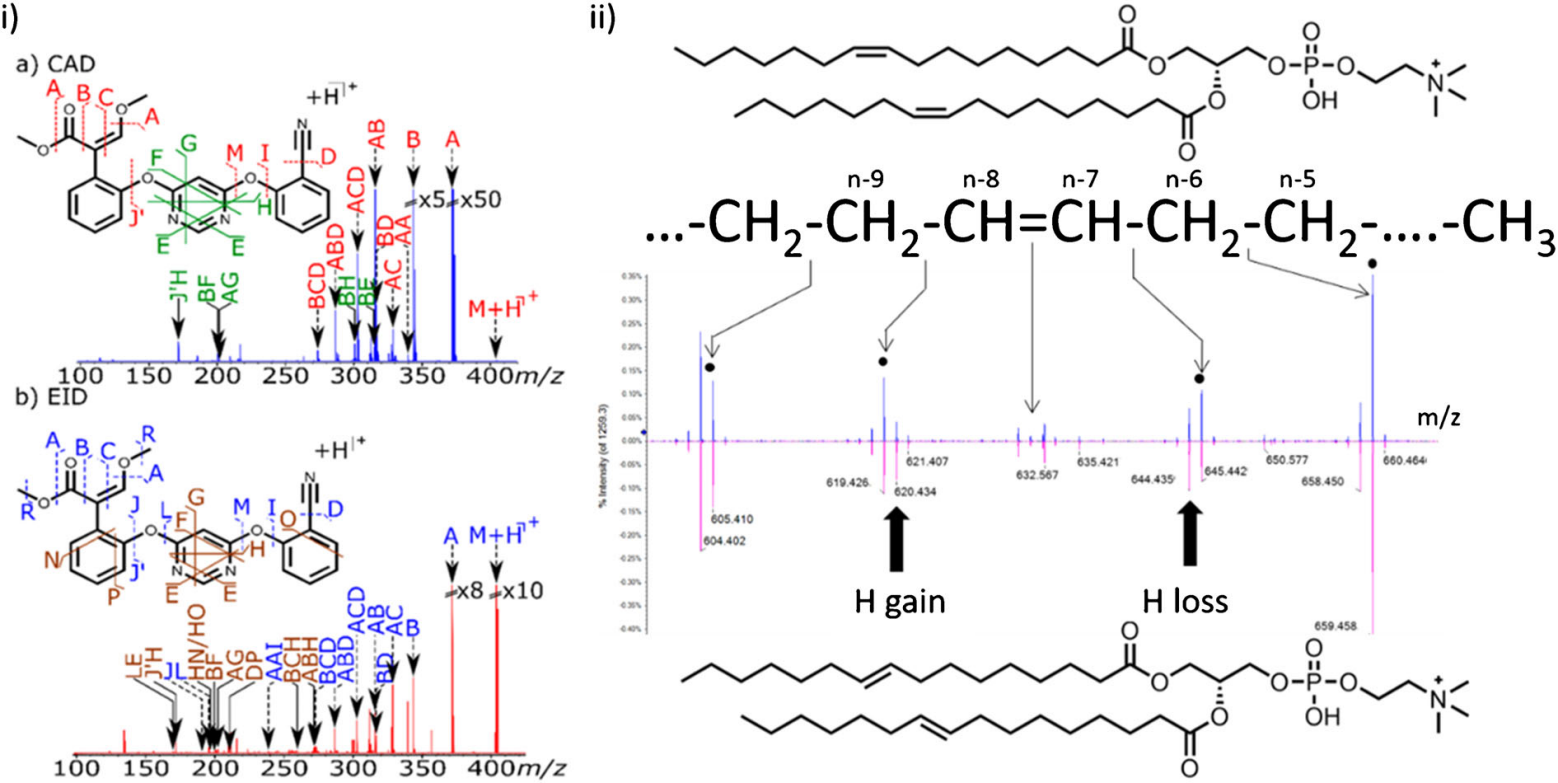
(**i**) (a) CID-MS^2^ and (b) EID-MS^2^ of azoxystrobin. Some assigned cleavage sites and corresponding signals are labeled. Reprinted with permission from [42], copyright 2020 American Chemical Society. (**ii**) EID-MS^2^ of protonated (blue) PC 16:1(9Z)/16:1(9Z) and (purple) PC 16:1(9E)/16:1(9E). Adapted with permission from [43], copyright 2017 American Chemical Society

For example, in one of the first studies that compared CID and EID, Lioe and O’Hair investigated protonated amino acids (AAs), singly charged Trp-containing dipeptides, and dimerized tripeptides [41]. The authors used comparable CID and EID activation settings to fragment [AA + H]^+^. Resulting CID spectra were dominated by neutral loss of NH_3_ and [H_2_O + CO]. In contrast, EID at 23 eV of [AA + H]^+^ yielded numerous fragment ions not observed in CID. The authors proposed that these additional fragmentation pathways are linked to electronic excitation of aromatic moieties as well as atomic hydrogen ejection followed by extensive fragmentation. Especially the observation of [M]^•+^ signals due to atomic hydrogen ejection suggests that EID fragmentation mechanisms are linked to fragmentation events under EI conditions. These proof-of-concept experiments demonstrated that EID and CID fragment ions can differ and that the additional EID fragment ions facilitate analyte identifications. In a recent study by Marzullo et al., the benefits of EID for metabolite structure elucidation for a set of seven agrochemicals were impressively showcased [42]. A representative example comparing CID (or collision-activated dissociation, CAD) and EID mass spectra of protonated azoxystrobin, a commonly used fungicide, is shown in Fig. 2. Although mainly ester and ether linkages cleave upon CID, the EID spectrum contains fragment ions associated with dissociation of virtually all segments of the molecule, including dissociation of aromatic moieties (N, P, O). The number and identity of the resulting EID fragment ions enabled assignment of the substitution patterns of all aromatic residues as well as identification of all compound moieties. The concept of structure identification with EID can even be extended to shotgun injection of complex mixtures when employing Fourier-transform ion cyclotron resonance (FT-ICR) instruments that enable two dimensional (2D) MS^2^ experiments. As demonstrated by the same research group, 2D MS^2^ of the mixture of the same seven agrochemicals creates a precursor *m*/*z* versus fragment *m*/*z* versus intensity counter plot containing wealth of structural information for structural annotations [44]. EID has been employed to study not only agrochemicals but also multiple exogenous and endogenous metabolites. For example, Mosely et al., Lopez-Clavijo et al., and most recently Ducati et al. utilized EID to investigate the fragmentation of positively charged pharmaceuticals [45–47]. In all studies, an increased number of fragment ions upon EID compared to CID were detected that often provided complementary information for structure identification. Additionally, the studies consistently showed that EID fragmentation patterns can depend on charge carrier identity. Moseley et al. found that protonated ions and ammonium adducts result in more EID fragment ions than sodium or potassium adducts, whereas Ducati et al. showed that especially EID of [M + Na]^+^ and [M + K]^+^ precursors yielded most fragment ions aiding compound identification. In addition to investigating positively charged xenobiotics, EID allows to dissociate deprotonated metabolites and peptides [48–50]. Nguyen et al. compared CID and EID results for deprotonated mononucleotides [50]. Unlike CID, EID tandem mass spectra of these metabolites contained cross-ring cleavage fragment ions and the authors were able to link these unique products of mononucleotide activation to hydrogen deficient radical anions formed upon electron–anion interactions. Tandem MS via EID has also provided rich fragmentation patterns for protonated ions of natural products [51–53]. In a series of experiments Chan and co-workers utilized EID to study carbohydrate containing metabolite ions [52, 53]. They, for example, were able to distinguish ganglioside isomers when using sodiated or deprotonated ions. But the makeup of other lipids can also be investigated with EID. One of the first studies that used EID to deduce lipid structures was authored by Yoo and Håkansson [54]. The authors used a FT-ICR MS to fragment [FA + Mn–H]^+^ lipid ions with EID, thereby pinpointing DB positions. Baba et al. implemented EID on a triple quadruple instrument significantly boosting sample throughput thereby enabling investigations of numerous lipid classes [43, 55–57]. With this setup, the authors investigated glycerophospholipids [57], sphingolipids [55], and triglycerides [56] demonstrating that FA composition, lipid head group, most abundant *sn*-isomers, and DB positions are available from EID fragmentation patterns. Some other advanced tandem MS tools provide the same structural information for lipids but only EID is able to distinguish cis/trans-isomers of DBs as shown in Fig. 2. In the zoom-in part of the EID spectrum of PC 16:1(9Z)/16:1(9Z) (blue) and PC 16:1(9E)/16:1(9E) (purple) fragment ion signals associated with cleavage of carbon–carbon bond in close vicinity to the DB are shown [43]. The relative signal intensities of features with *m*/*z* 620 and *m*/*z* 644 formed due to hydrogen gain and hydrogen loss differ between cis- and trans-isomers, respectively. The authors rationalized this finding by proposing a transiently formed biradial species due to EID excitation of the DB, which subsequently dissociates. Because energetics of intramolecular rearrangements differ between cis- and trans-isomers, the relative abundance of the corresponding fragment ions is diagnostically changed. At the moment, this is the only available tandem MS method to distinguish lipid DB cis-/trans-isomers. Despite the great promise of EID for structure identification, fragmentation efficiencies, i.e., the summed signal intensities of all fragments relative to the sum of all fragments and precursors, is typically lower than in CID and is affected by the analyte identity currently limiting widespread use of this advanced tandem MS method.

## Photon-based fragmentation

Instead of activating analyte ions with electrons, photons can be used to trigger ion dissociation in the gas-phase. Light of all wavelengths could be used for this purpose but the absorption as well as dissociation characteristics of metabolites and the availability of appropriate light sources determine the choice of appropriate wavelength regimes for tandem MS applications. In particular, most tandem MS applications are restricted to the IR and the UV region of the electromagnetic spectrum as the large majority of metabolites as well as lipids exhibit intense absorption features and corresponding table-top light sources are commercially available. Functional groups such as carbonyls, conjugated systems, benzene moieties, and aromatic heterocycles with their pronounced n → π* as well as π → π* transitions typically absorb UV light between 190 and 320 nm. With the aid of nanosecond pulsed solid-state and gas-phase laser systems, which offer energies up to 150 J/pulse and often operate at 157 nm, 193 nm, 213 nm, 248 nm, 266 nm, 337 nm, 351 nm, and 355 nm with repetition rates between 10 and 5000 Hz, many metabolite and lipid ions are readily activated in the gas phase. In the IR region especially O–H, N–H, C=O, and P–O, valence vibrations or ubiquitous deformation modes are targeted by employing fixed or tunable-wavelength optical parametric oscillators/optical parametric amplifiers (OPO/OPA) and high-power CO_2_ laser systems (10.6 μm).

**Scheme 1:**
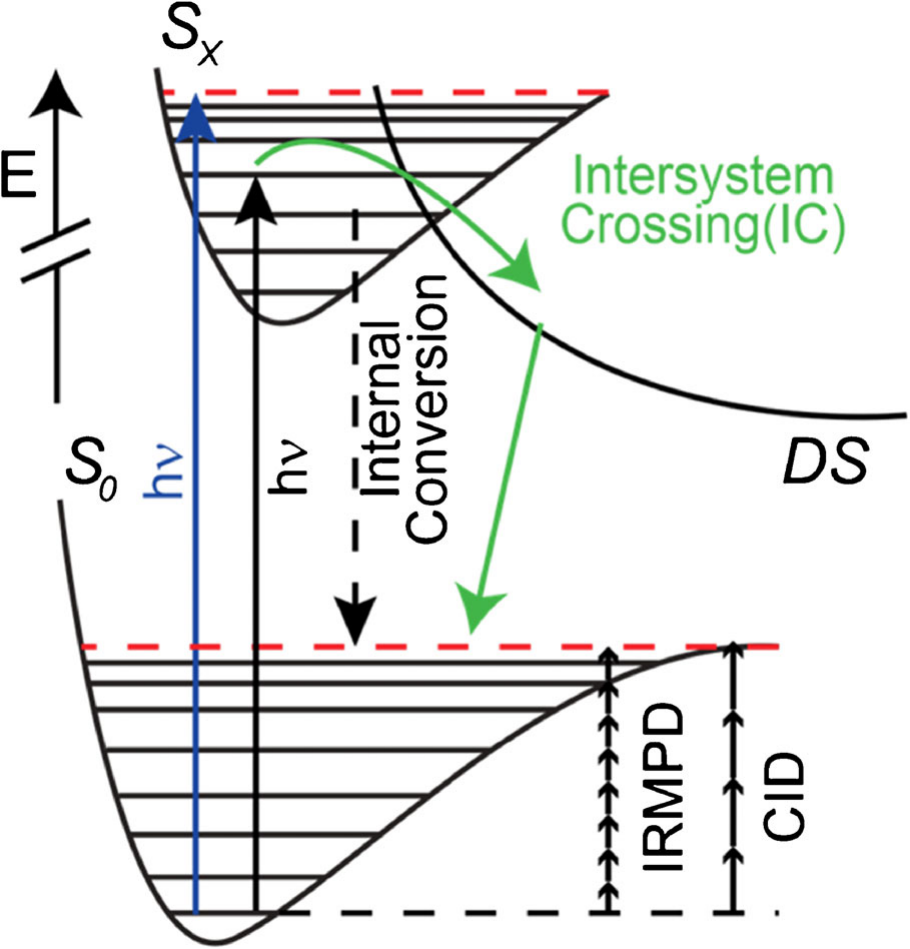
Jablonski diagram schematically showing ion activation and dissociation energetics by UV/IR photons or gas collisions

In the case of IR radiation, the corresponding tandem MS method was termed IR multiple photon dissociation (IRMPD) and was first explored for the use in biomolecular MS by McLafferty and co-workers [58]. The name stems from the fact that a single IR photon (11 kJ/mol for 10.6 μm) is not sufficient to cause bond rapture in most biologically relevant molecules (amide bond dissociation enthalpy typically ~ 335 kJ/mol) and consecutive IR photon absorption is required to trigger ion dissociation. The energetics of the process are schematically shown in Scheme 1. One IR photon is absorbed by a vibrational mode of a gas-phase ion (black arrow), thereby increasing the inner energy of the system. In order to overcome the dissociation threshold in the electronic ground state (S_0_, red dashed line), multiple absorption events must take place. A more detailed discussion on this topic and IRMPD is provided by Polfer and Oomens [59]. From Scheme 1, the energetics of IRMPD and CID appear to be similar. One big difference between CID and IRMPD is that the choice of IR laser or IR transition enables some degree of selectivity during tandem MS. This was, for example, demonstrated by Crowe and Brodbelt [60]. The authors showed that phosphorylated peptides are more efficiently dissociated compared to non-phosphorylated compounds in IRMPD, whereas the tandem mass spectra of both compound classes with CID were similar. As commercial mass spectrometers, such as FT-ICR MS, have been equipped with IRMPD units, numerous groups have used IRMPD to fragment analyte ions. For lipids and metabolites, however, the number of studies employing fixed wavelength IRMPD are rare. Mostly metabolites with phosphate groups and secondary plant metabolites have been investigated with IRMPD. For example, Yoo and Håkansson used IRMPD to fragment phosphorylated metabolites observing increased fragmentation efficiency for metabolites with the most number of P–O bonds [48]. In a study by Bianco et al., IRMPD was utilized to fragment glucosinolates extracted from *Capparis spinosa* [61]. Glucosinolates consist of sulfated N-hydroxy thioamides that are glycosidically linked via sulfur to a carbohydrate. IRMPD tandem MS allowed to obtain structurally diagnostic fragment ions suggesting that S=O groups efficiently absorb 10.6 μm photons.

This is also consistent with the recent observation that S-sulfonylated peptides readily fragment upon IRMPD [62]. In other studies of plant metabolites that lacked functional groups that efficiently absorb 10.6 μm IR radiation, IRMPD and CID fragment ions and fragment ion intensities were similar [63]. Consequently, fixed wavelength IRMPD does often not provide complementary fragment ions to CID unless metabolites contain P–O or S=O groups.

**Fig. 3:**
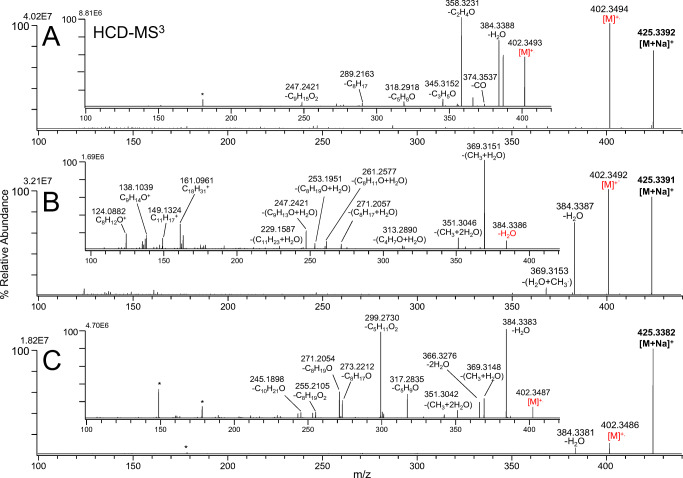
**A**, **C** MS^3^ and **B** MS^4^ of sodiated **A** 4β-OH cholesterol, **B** 7a-OH cholesterol, and **C** 25-OH cholesterol employing 213 nm UVPD followed by HCD allow to distinguish steroid isomers. Reprinted with permission from [68], copyright 2020 Elsevier B.V.

This is in contrast to activation methods that rely on UV irradiation of gas-phase ions termed UV photodissociation (UVPD). UVPD for tandem MS of biomolecules was first reported by Bowers et al. in 1984 [64] but only the broad availability of high-performance MS instruments such as FT-ICR and orbital trapping mass spectrometers in the last two decades propelled UVPD from niche analytic applications to an emerging tandem MS tool in routine omics workflows. In UVPD, gas-phase ions are excited from an electronic ground state into an excited electronic state. Fragment ion formation often only requires single photon absorption or consecutive absorption of a few (less than five) UV photons due to photon energies close to or above ion dissociation energies. Single photon absorption from the S_0_ to the S_x_ state (blue and black arrows) and related photochemical processes are schematically shown in Scheme 1. In the excited electronic state, a multitude of photochemical processes can lead to dissociation of analytes. Relaxation via fluorescence and phosphorescence [65, 66] can also occur but these processes will be excluded in the discussion because they will not yield fragment ions. Internal conversion leads to conversion of excess energy into the rovibrational heat bath of the activated molecule thereby potentially overcoming bond dissociation enthalpies and causing fragmentation. Therefore, fragmentation pathways similar to CID can be accessed but fragment ions requiring higher activation energies than CID are released too. Alternative activation scenarios are UV excitation of analyte ions above the dissociation threshold in the excited electronic state (Scheme 1, blue arrow) or intersystem crossing to dissociative states (DS). Due to this multitude of UV-trigged dissociation mechanisms, UVPD tandem mass spectra of complex biomolecules often contain a large number of fragment ions. Some of them are also observed in CID experiments but additional UVPD-specific product ions can aid structure identification. For this reason, a multitude of research groups have employed UVPD, mostly using 157 nm, 193 nm, and 213 nm laser irradiation, to identify the structure of metabolites. For more details about UVPD in bioanalytical science, especially in proteomics, the reader is referred to the excellent review by Brodbelt and co-workers [67]. In two recent studies, the commercially available 213 nm UVPD unit for the Orbitrap Fusion Lumos Tribrid (Thermo Fisher Scientific) was used to study organic micro-pollutants (OMPs) and steroids. In particular, West and Reid utilized the multistage tandem MS capabilities of the instrument to generate radical cations from sodiated steroids via 213 nm UVPD and subsequently activate radical ions via vendor-specific CID (higher-collisional dissociation; HCD) [68]. Corresponding MS^n^ tandem mass spectra for isomeric species 4β-OH cholesterol, 7α-OH cholesterol, and 25-OH cholesterol are shown in Fig. 3. After UVPD and HCD activation, diagnostic fragment ions, e.g., *m*/*z* 345.31 (4β-OH cholesterol), *m*/*z* 313.28 (7α-OH cholesterol), *m*/*z* 299.15 (25-OH cholesterol), are present that are not released upon HCD. In another study by Panse et al., single-stage 213 nm UVPD afforded more structure diagnostic fragment ions for water-relevant OMPs than CID, thereby improving OMP characterization in direct infusion measurements [69]. Although UVPD has not been widely used to study water-solvable metabolites, an extensive body of work that documents the ability of UVPD to structurally characterize oligosaccharides, glycolipids, sphingolipids, and glycerophospholipids has been reported. For example, Reilly and co-workers showed that 157 nm UVPD increases the number of cross-ring cleavages for positively charged oligosaccharides compared to CID facilitating identification of carbohydrate linkage patterns [70]. The increase of cross-ring cleavage efficiency as well as retention of labile groups compared to CID in positive as well as negative ion mode was also reported by Racaud et al. and Brodbelt and co-workers employing ~ 220 nm and 193 nm laser light to fragment heparin-derived disaccharides [71, 72] and glycosaminoglycans [73], respectively. This increased abundance and number of cross-ring product ions upon UVPD compared to CID not only is beneficial for isolated oligosaccharides but also enables structure interrogation of glycol- and saccharolipids.

**Fig. 4:**
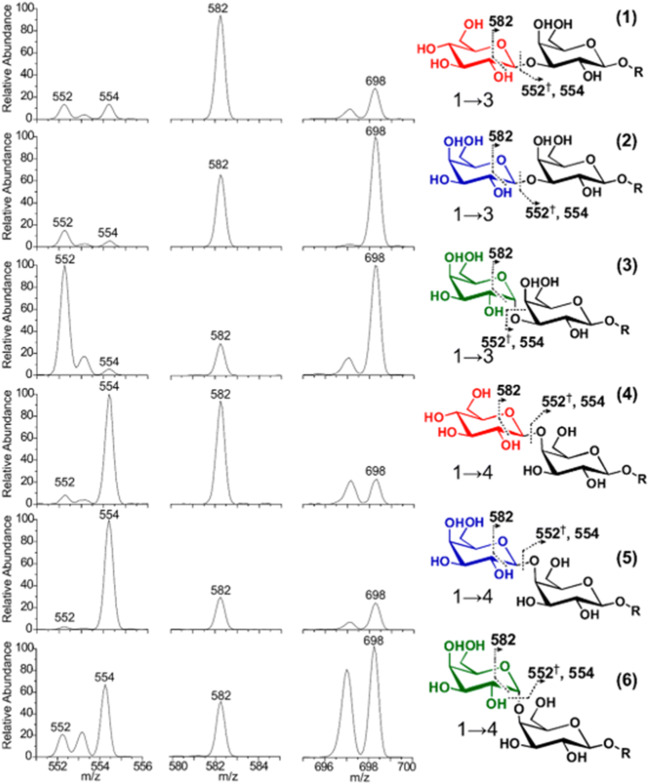
Selected mass spectrometric signals obtained by RDD of sodiated disaccharide isomers that allow isomer discrimination. Reprinted with permission from [87], copyright 2018 American Chemical Society

In particular, the Brodbelt group used 193 nm UVPD to dissect bacterial lipid A compounds as well as gangliosides [74–76]. In addition to product ions from the saccharide moieties of these compounds, diagnostic ions for FA side chains and the sphingoid base helped to extensively characterize the structure of these complex lipids. Follow-up studies showed that optimized experimental settings enable identification of DB positions [77, 78], *sn*-isomers [77, 79, 80], hydroxylation sites as well as linkages [81], and FA branching/cylclopropanation [82] sites in glycerophospholipids and sphingolipids when using 193 nm or 213 nm UVPD. All these studies showed that UVPD results in extensive metabolite and lipid fragmentation, thereby enabling annotation of structural details not accessible with CID methodologies. The biggest caveat of UVPD, however, is that the absorbance of functional groups at UVPD wavelengths ultimately affect fragmentation efficiency as well as fragmentation pathways. Therefore, fragment ion signals are typically much lower in UVPD compared to CID. In order to control UV absorption, selective UVPD of covalently or non-covalently linked chromophores with well characterized absorption and dissociation characteristics can be utilized to exert more control over UV-triggered processes. The most prominent strategy is radical-directed dissociation (RDD) pioneered by Ryan R. Julian. By installing an iodo-benzene moiety on analytes, 266 nm UVPD homolytically cleaves the C–I bond due to rapid intersystem crossing of electronically excited benzene groups to a dissociative nσ* state creating a benzene radical. CID of iodine-deficient radical ions leads to structurally diagnostic fragments [83]. This method, originally developed for peptides and proteins, was adapted by the groups of Julian and Blanksby to study lipid as well as oligosaccharide ions. In RDD studies of these analytes, DB position, FA branching positions and diastereomers of FAs [83, 84], GPs [85], and glycerosphingolipids [86] were identified by employing custom-made iodine-containing chromophores. But controlled radical delivery via RDD also allows differentiation of oligosaccharides as demonstrated by Riggs et al. and shown in Fig. 4 [87]. By irradiating derivatized disaccharides with 213 nm laser light, the authors were able to obtain characteristic UVPD/CID fragmentation patterns. The relative fragment ion intensities differs between all disaccharides allowing to distinguish isomers, including anomers. Even though RDD effectively enables on-demand generation of reactive precursor ions, the random nature of subsequent processes complicates controlled radical delivery to structural elements of interest, a bottleneck of the method to be addressed in the future.

## Ion/molecule and ion/ion reactions

Often the functional groups or the charge state of analytes and associated energetic demands prevent formation of structurally diagnostic product ions via CID. Although electron-based and light-based fragmentation strategies rely on different means of ion activation than gas collisions, reactions of analyte ions in the gas phase can affect dissociation energetics or modulate the ion charge, thereby facilitating formation of informative product ions. Therefore, methods that react ions with neutral molecules or other ions in the gas phase have been developed. These ion/ion and ion/molecule reaction schemes have, for example, been reviewed by McLuckey and co-workers [88, 89] and Osburn and Ryzhov [90], respectively. Originally developed for applications in the field of proteomics, such as the commercially available electron transfer dissociation (ETD) method [91], numerous recent investigations have targeted metabolites.

**Fig. 5:**
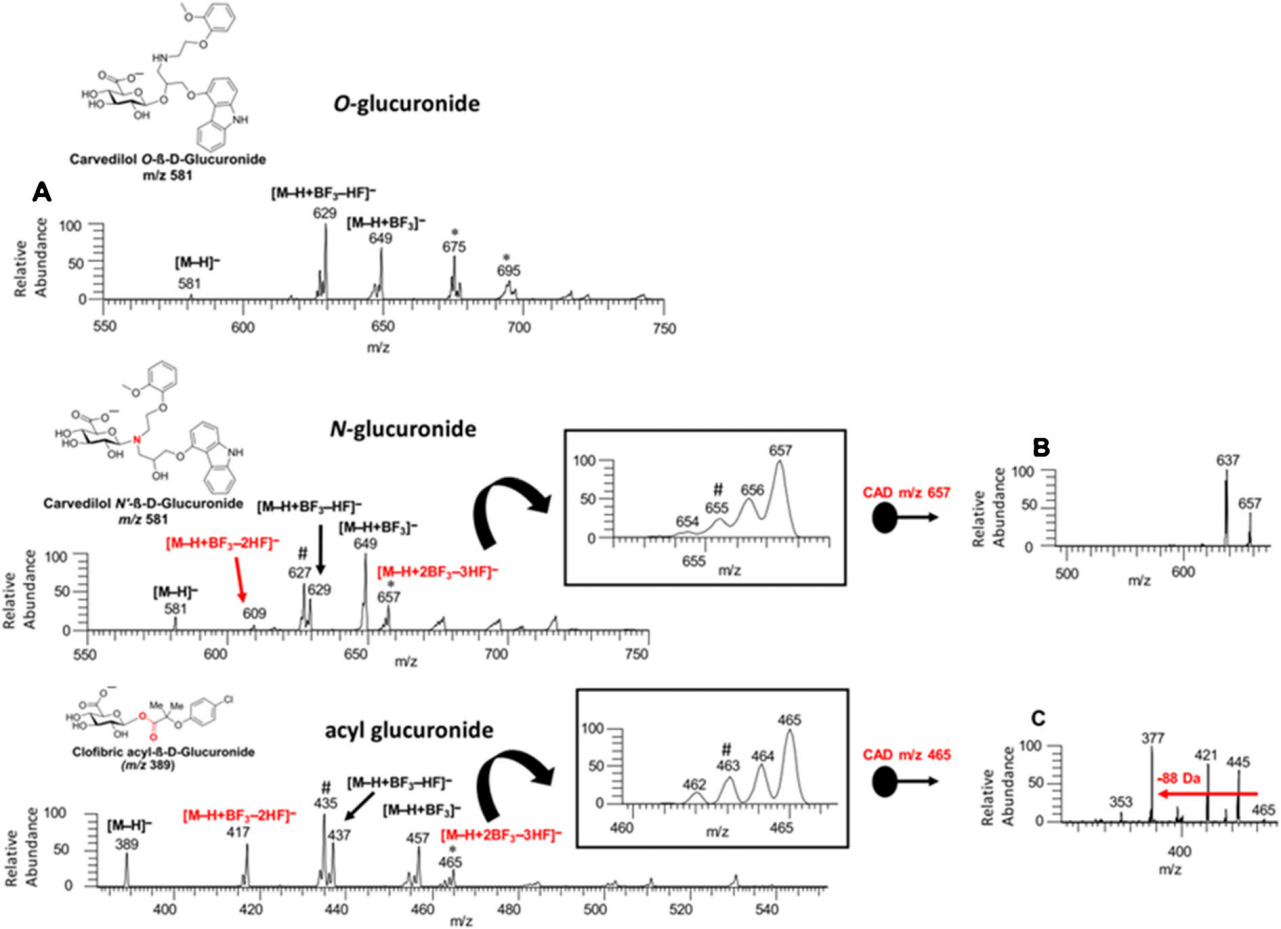
Ion/molecule reactions between BF_3_ and different glucuronides. Although O-glucuronides do not form ions with three neutral losses of HF, ion/molecule spectra of N- and acyl-glucuronides contain these diagnostic signals. The latter two glucuronides are distinguished by CID of ions with three HF losses. Reprinted with permission from [100], copyright 2019 American Chemical Society

In ion/molecule reactions, gaseous neutral molecules are injected into high-pressure regions of a mass spectrometer, such as the inlet system, the collision cell, or an ion trap, in order to interact with analyte ions. Ion/molecule reactions are often specific for one or a small number of functional groups making them perfectly suited to investigate structural changes of these specific structural elements. For example, Blanksby and co-workers utilized ozone to target DBs in lipids and metabolites [92]. By reacting O_3_ with analytes, DBs are selectively transformed into ozonides that spontaneously dissociate to yield Criegee and aldehyde fragment ions with structure-specific *m*/*z* values. This method was termed ozone-induced dissociation (OzID) and can reveal the location of lipid DBs in MS^2^ and *sn*-isomers in MS^3^ experiments. In a recent study, Marshall et al. employed OzID to identify relative FA positions in TGs [93]. For this purpose, [TG + Na]^+^ ions were activated by CID and resulting FA loss ions most likely contain a 1,3-dioxolane ring with a ring-adjacent DB formed by one of the remaining FAs [94]. Subsequent OzID preferentially dissociated the newly formed ring-adjacent DB revealing neighboring FAs. Combined with the ability to identify DBs in OzID MS^2^ experiments, this powerful analytic platform has been utilized to structurally characterize SLs [95], GLs [93, 95], FAs [96], and GPs [95]. However, ion/molecule reaction yields are often limited by the partial pressure of the regent gas or can contaminate the mass spectrometer if reactions are not carefully optimized. Other gas-phase ion/molecule reactions than reactions with O_3_ have been used to study metabolites. Another strategy is to react ions with highly reactive species such as hydrogen/oxygen atoms or hydroxyl radicals. By dissociating hydrogen and different oxygen-containing compounds via a heated tungsten filament and via a plasma generator, respectively, Takahashi et al. were able to interrogate lipid ion structures [97]. Reaction of lipid ions with hydrogen atoms and radical oxygen species resulted in formation of metastable lipid radical ions revealing DB positions and *sn*-isomer abundances upon activation. In other experiments, Kenttämaa and co-workers have shown that BF_3_ or trimethoxymethylsilane can track the presence of adjacent functional groups in glucuronide [98] or sulfone/carboxylic acid/sulfonamide [99] groups after ion/molecule reactions, respectively. For example, Niyonsaba et al. investigated negatively charged drugs ion/molecule reaction mass spectrometry. Although CID did not allow unambiguous differentiation between acyl-, O-, and N-glucuronides, ion/molecule reaction of analyte ions with BF_3_ yielded diagnostic reaction products as shown in Fig. 4 [98]. O-Glucuronides did not result in product ions with more than one HF loss, in contrast to N- and acyl-glucuronides that readily lost up to three HF units. This ion/molecule reaction not only allowed to distinguish isomeric O- and N-glucuronides but also subsequent CID of ions with attached BF_3_ and loss of three HF units revealed the characteristic loss of C_2_H_2_O_2_BF (88 Da) only for acyl-glucuronides (Fig. 5). This concept of ion/molecule reactions has been extended to other reactive species [100] and used to study drugs and drug metabolites [101, 102].

**Fig. 6:**
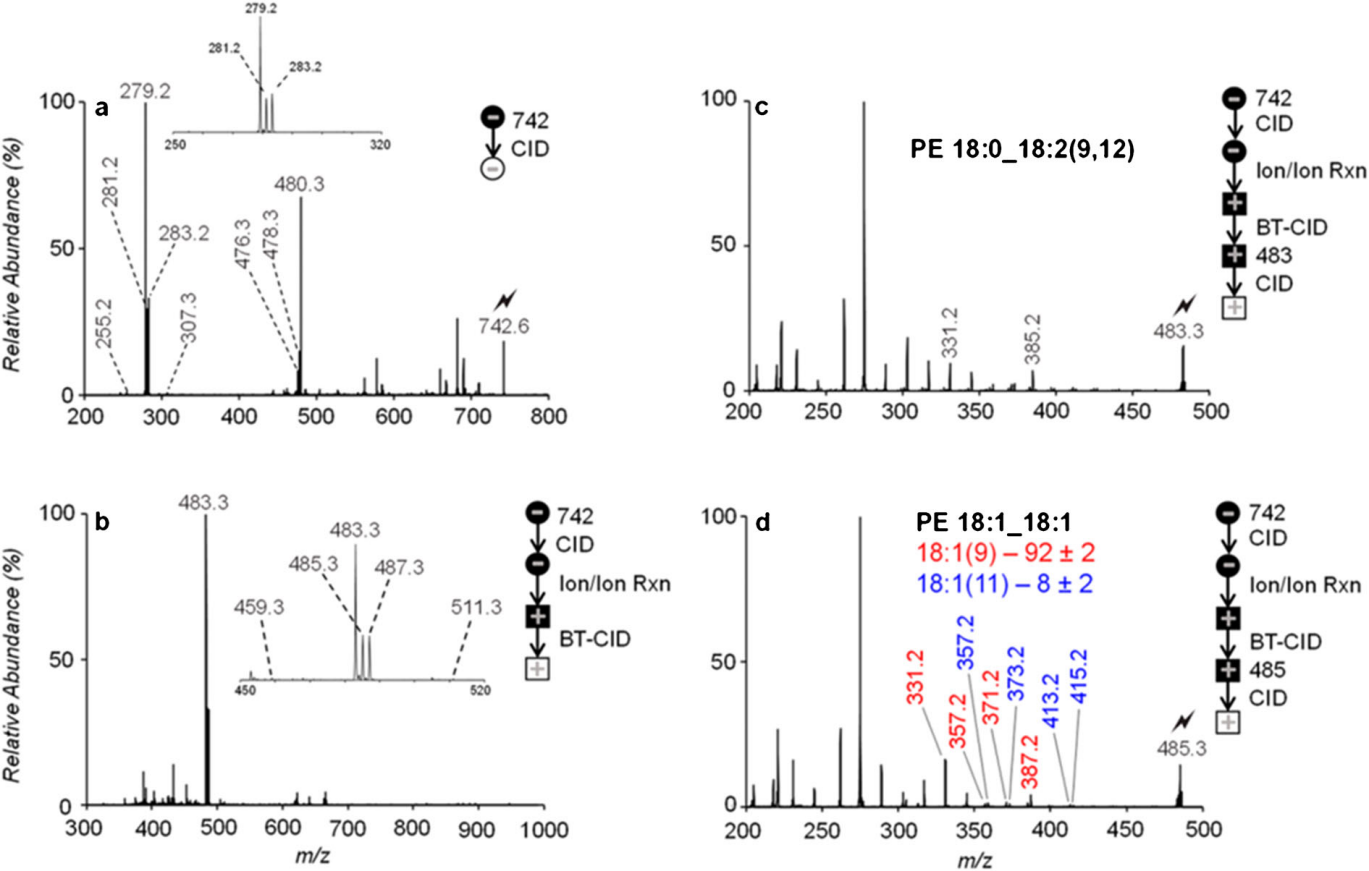
Analysis of PE 36:2 from human plasma with ion/ion reactions. Deprotonated PE 36:2 reveals head group and FA composition upon **a** CID, **b** FA attached to [MgPhen_3_]^2+^ are formed after ion/ion reactions and beam-type CID. The resulting positive ions of **c** FA 18:2 and **d** FA 18:1 enable DB position assignment upon CID. Reprinted with permission from [106], copyright 2020 American Chemical Society

Not only reactions between ion and molecules are feasible but also ion/ion reaction, mostly performed in ion traps, can yield diagnostic fragments. Although reactions between ions of opposite charges exhibit large CCSs due to the long range of attractive Coulombic interactions, charge transfer dissociation (CTD) enables reactions between positively charged inert gas ions and negative as well as positive analyte ions. By accelerating He ions to 6 keV, ion/ion collisions and most likely oxidation of analytes to radicals result in activated analytes prone to fragment [103]. CTD yields tandem mass spectra with a large number of fragment ions not observed with CID enabling structural characterization of GPs [104] and oligosaccharides [105]. Reactions between low-energy ions of opposite charges are currently employed by numerous groups, with the McLuckey group pioneering many of these ion/ion reactions especially in the context of lipid analysis. By simultaneously trapping positively double charged alkaline earth metal trisphenanthroline complexes (MPhen_3_^2+^) with negatively charged lipid ions in an ion trap mass spectrometer, charge-mediated MPhen-lipid complexes form that are positively charged [106]. Thus, these experiments allow to perform CID in negative ion mode to reveal FA composition (Fig. 6a). After MPhen complexation and charge-inversion (Fig. 6b), CID unveils DB positions as showcased for PE 36:2 from human plasma extract in Fig. 6c, d. In this particular case, PE 36:2 was shown to comprise of PE 18:0_18:2(9,12) (Fig. 6c), PE 16:0_20:2, and PE 18:1_18:1 with DBs at position 9 and 11 (Fig. 6d). This charge-inversion strategy was also used to study the structures of FAs [107], CLs [108], and glycosphingolipids [109] demonstrating the broad scope of compound classes addressable with this advanced tandem MS tool.

## Chemical derivatization prior to tandem MS

**Scheme 2:**
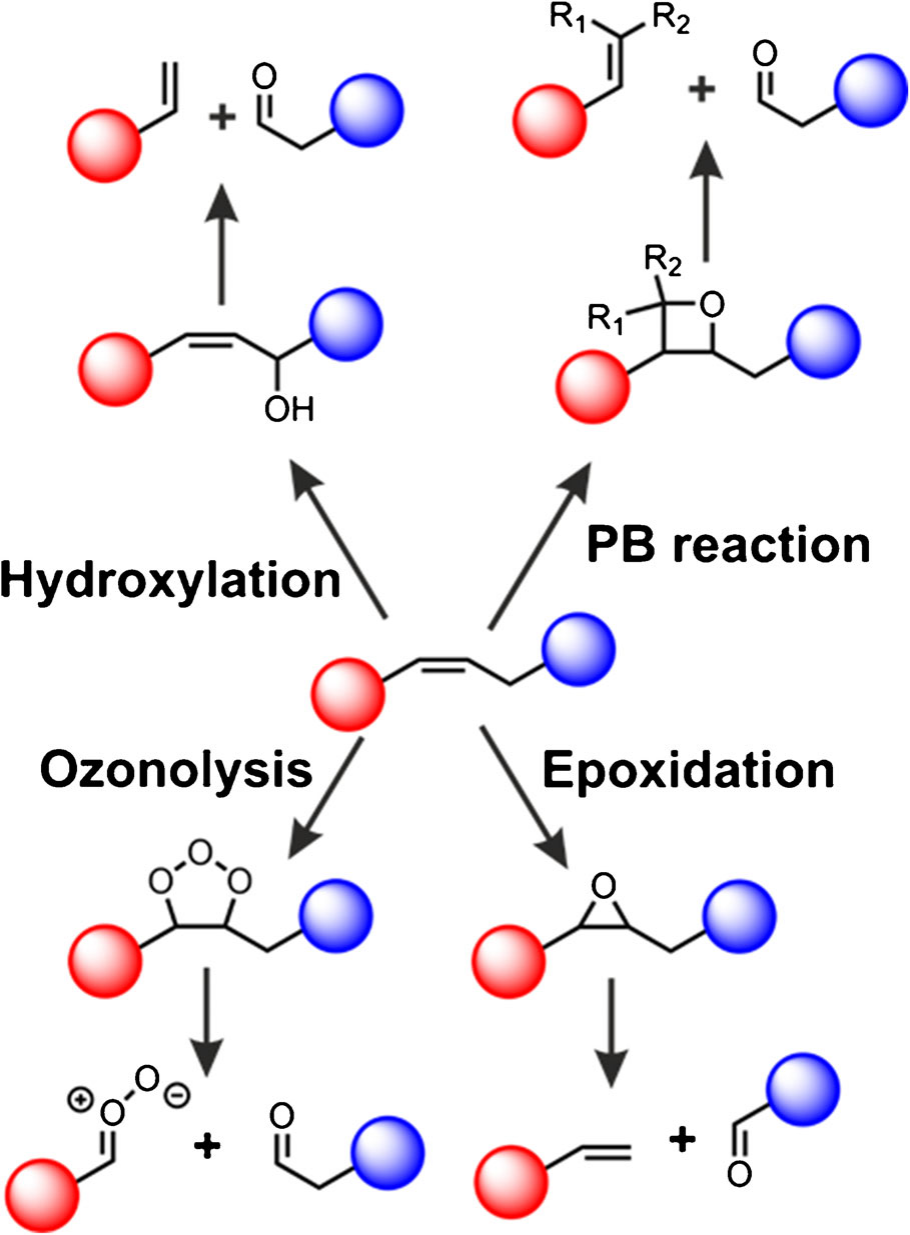
Derivatization strategies targeting lipid and metabolite DBs and enabling DB position assignment after tandem MS of product ions

Although chemical derivatization is routinely employed to increase analyte stability, install isotopic markers, or improve ionization yields in metabolomics and lipidomics for both LC-MS and MSI [13, 110–113], derivatization reactions that improve structural characterization of analytes without suffering consequences of complicated sample preparation steps were rare until recently. However, the discovery by Ma and Xia [114] that light-induced Paternò–Büchi (PB) reactions can aid DB position assignments with relative ease and with inexpensive equipment has sparked the interest in chemical derivatization strategies that enable structure elucidation. In particular, most recent derivatization strategies target lipid DBs or adjacent carbon atoms and are based on PB [114–122], epoxidation [123–125], ozonolysis [126], or hydroxylation reactions [127] (Scheme 2).

**Fig. 7:**
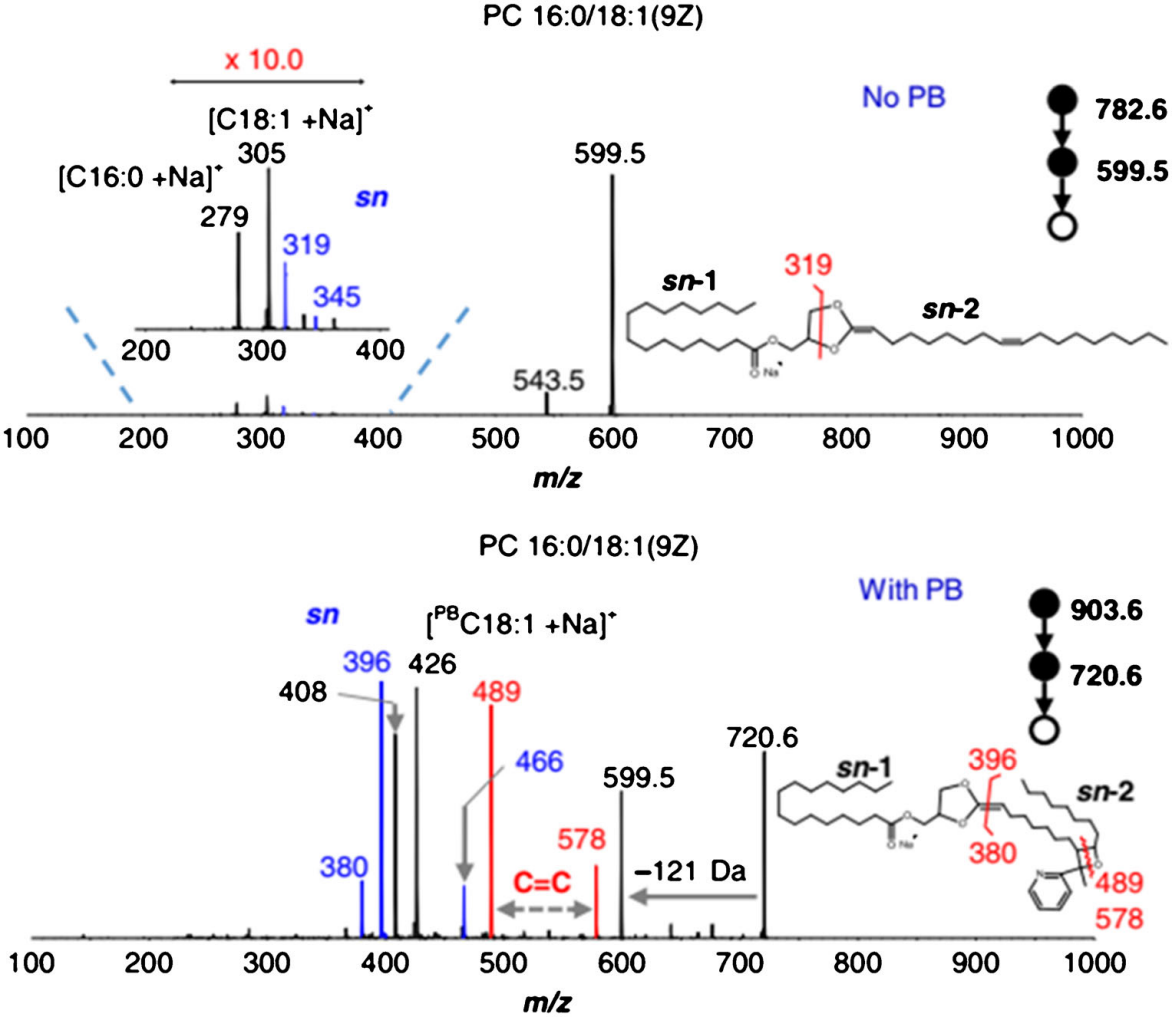
Comparison of CID-MS^3^ of sodiated PC 16:0/18:1(9Z) (upper) before and (lower) after PB functionalization with 2-acetylpyridine. DB positions (red signals) and *sn*-isomers (blue signals) are only confidently identified after PB functionalization. Adapted with permission from [121], copyright 2020 the authors

In PB reactions between UV light-activated carbonyl compounds and lipid DBs, oxetanes are formed. Reactions are typically performed within the ion source with 254 nm UV light transforming up to 80% of unsaturated lipids in less than 1 min into corresponding reaction products [115, 128]. Intact transfer of these oxtanes into the gas-phase followed by CID yields product ions diagnostic for DB positions (Scheme 2). Since the first experiments with acetone as PB-reactive compound, next-generation PB compounds that increase reaction yields and ionization efficiencies compared to acetone have been utilized in PB workflows or compounds that require visible light to start the PB reaction have been identified [129]. For example, Esch and Heiles as well as Cao et al. have explored acetylpyridine (acpy) compounds for PB reactions in lipidomics [116, 121]. The use of 2-acpy not only results in efficient PB product formation but also allows to distinguish DB position as well as *sn*-isomers as shown in Fig. 7 for PC 16:0/18:1(9Z). Although CID-MS^3^ of sodiated PC 16:0/18:1(9Z) mainly yields FA fragment ions (Fig. 7, upper), PB reaction followed by CID-MS^3^ results in abundant product ions that pinpoint the DB position (red) and reveal *sn*-isomers (blue) (Fig. 7, lower). PB reactions with acpy and other carbonyl compounds have been used not only to analyze standards but also to investigate complex lipid extracts from body fluids, cells, or tissues revealing DB positions and sometimes *sn*-isomers for CEs [116, 130], FAs [116], GPs [121], and SLs [131]. Recently, PB methods have been extended to also target small FA metabolites [117–119] or have been adapted to benefit other advanced tandem MS tools such as UVPD [115] or ion/ion reactions [122].

Other derivatization methods install different forms of reactive oxygen species close to or at lipid DBs. These methods do not require specialized or expansive solution additives to functionalize analytes but the mass of resulting reaction products is often only shifted by + 16 Da, + 32 Da, or + 48 Da. This can potentially cause overlap with isobaric lipid species in complex extracts. One example is the simple yet powerful epoxidation strategy developed by Li and co-workers [123]. By in-solution mixing of lipids with meta-chloroperoxybenzoic acid, unsaturated components of the extract are efficiently transformed into epoxides. After reaction to single or multiply epoxidated compounds, CID-MS^2^ of reaction products liberates fragment ions diagnostic for DB positions (Scheme 2). Epoxidation of lipids can also be achieved by low-temperature plasma treatment of acetone-containing lipid solutions transiently forming acetone peroxide [125], upon ionization in a triboelectric nanogenerator [132], or on-demand electrochemical epoxidation in ESI sources [124]. However, the downside of many derivatization strategies developed for tandem MS are unwanted side reactions such as Norrish-type reactions or overoxidation, which lower overall reaction yields and complicate resulting mass spectra. For this reason, derivatization strategies are often complex to implement in analytic workflows and more work is still needed to combine ease-of-use with analytic performance.

## MS-based spectroscopy of lipids and metabolites

Despite the increased number of structure diagnostic fragment ions provided by advanced tandem MS methods compared to classical CID, structure assignments merely rely on fragment ion identities or relative intensities. This limits the success of structure identification that requires specific bonds to dissociate compared to spectroscopic methods that probe molecular energy levels. Combining the potential of tandem MS with the ability to record spectroscopic data is, thus, highly desirable when attempting structural characterization of metabolites and lipids. IRMPD and UVPD already make use of vibrational and electronic excitations to obtain tandem mass spectra but are often performed with a single or a few wavelength settings, respectively. In recent years, affordable tunable table-top laser systems in the IR and UV became available that allow to perform wavelength-dependent IRMPD and UVPD investigations. By recording IRMPD or UVPD tandem mass spectra as a function of IR or UV excitation wavelength and monitoring fragment ion intensities, IRMPD or UVPD spectra are reconstructed, respectively. Numerous studies have demonstrated that the resulting spectra often closely resemble linear IR and UV spectra as long as the absorbed energy is sufficient to dissociate ions [59]. These spectra can be utilized to characterize analytes based on structurally diagnostic spectroscopic signatures. Because the effect of IR or UV light on analytes is indirectly probed by a mass spectroscopic read-out and not by the decrease of light intensity, all these methods are termed action spectroscopy. Originally used in physics and physical chemistry communities, the enormous potential for solving analytic problems has led to action spectroscopy methods being adapted for metabolomic and lipidomic workflows [133].

**Fig. 8:**
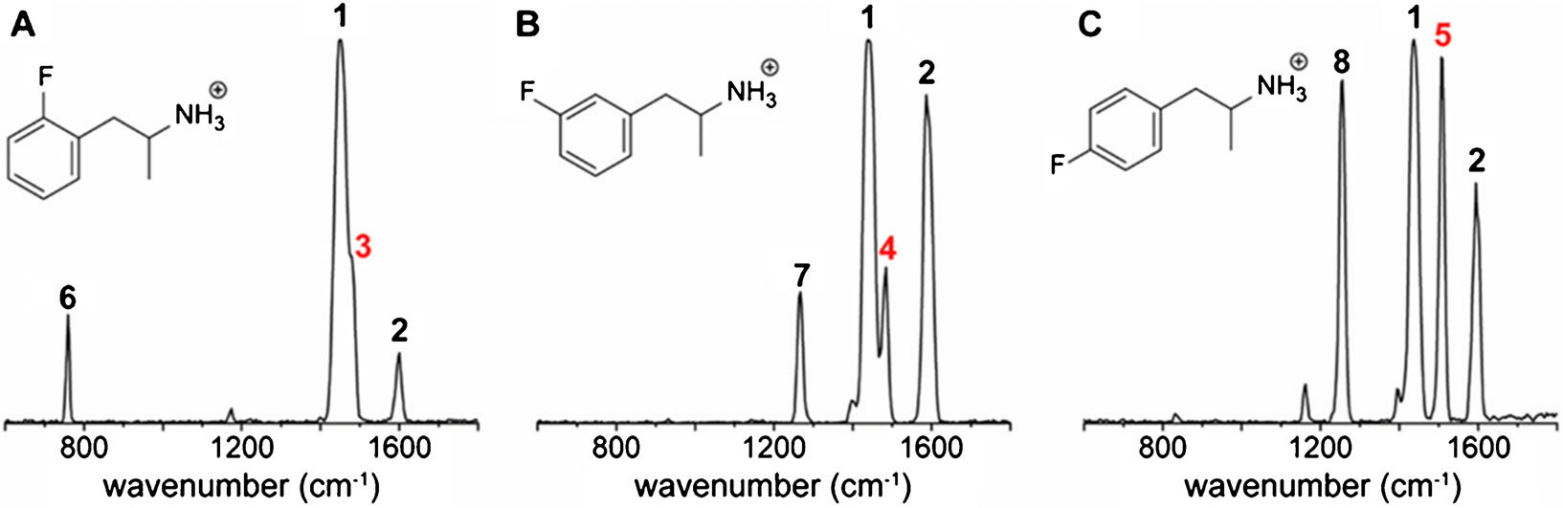
IRMPD spectroscopy of three fluoroamphetamine isomers differing only in the position of the fluorine moiety. Spectroscopic features, especially those labeled with 3–8 are structurally diagnostic. Reprinted with permission from [134], copyright 2020 American Chemical Society

In a recent study, Kranenburg et al. used IRMPD spectroscopy to distinguish fluoroamphetamine isomers [134]. These synthetic novel psychoactive substances have virtually identical EI mass spectra as well as IRMPD tandem mass spectra. In contrast, IRMPD action spectra of ESI-generated protonated ions are shown in Fig. 8. Although some spectroscopic bands have similar intensities and appear at similar wavenumbers (labels 1 and 2), characteristic features are obtained for every isomer indicating structure-specific vibrational modes of these molecules (labels 3–8). Assignment of these spectra to specific features is, for example, possible by comparing experimental results to quantum chemical simulations of vibrational modes. Another possibility is the development of databases and, similar to EI-MS or CID-MS^2^, implementing automated database searches and assignments. Another benefit of action spectroscopy experiments is the combination of sequential CID/IRMPD and IRMPD spectroscopy to structurally interrogate fragment or remaining precursor ions. As shown by Martens et al. [135] and von Geenen et al. [25], fragmentation of isomeric dicarboxylic acids and fluoromethamphetamines (Fig. 1) followed by IRMPD spectroscopy allows to deduce precursor and fragment structures. The concepts outlined for the discussed prototype compounds are readily adaptable to other pharmaceuticals [134] or oligosaccharides [136].

Cooling of gas-phase ions and/or attachment of inert gas molecules can further improve the ability to separate spectroscopic features by narrowing the width of spectroscopic bands albeit custom-made instrumentation is often required. For example, Mucha et al. [137] and Kirschbaum et al. [138, 139] used IR action spectroscopy of cooled ions to differentiate isomeric oligosaccharides and sphingolipids, respectively. Cold ion UVPD spectroscopy of isomeric ephedrines [140] and glycans [141] also revealed the potential of tandem MS performed with tunable-laser systems to distinguish isomeric metabolites and lipids. The biggest challenge for all advanced tandem MS methods and especially for spectroscopic tools is the integration into routine LC-MS^n^ and MSI workflows. This is because the chromatographic peak width of only couple of seconds (typically 2–120 s) complicates collection of spectroscopic signatures for a wide wavelength range. Additionally, analytic figures of merit and ease-of-use provided by LC-MS^n^ methods set standards currently not achieved by newly developed advanced tandem MS tools. Recent developments, however, push the limits of advanced tandem MS strategies attempting to establish these tools in MS-based lipidomic/metabolomic workflows as outlined in the next two sections.

## First LC-MS^n^ case studies

A large portion of modern lipidomic and metabolomic studies utilize the power of LC-CID-MS^n^ to minimize matrix effects and ion suppression, thereby accomplishing limits of detection (LOD) down to ng/mL [142]. Most of the advanced tandem MS methods described above, however, can be considered proof-of-concept studies that mostly rely on direct infusion measurements of authentic standards and selected complex mixtures. To progress from these method development studies towards routine high-throughput applications, advanced tandem MS tools must be adapted for LC-MS^n^ experiments. The challenges associated with the transition from direct infusion to LC experiments are multifaceted. For some of the structure-sensitive tools described above, fragmentation efficiencies are lower than for CID consequently increasing LODs and limits of identification for analytes. Other obstacles are associated with the ion activation time that is often longer than the chromatographic peak width, with the availability of these methods on commercial instruments, and/or with solution additives that are incompatible with LC systems.

**Fig. 9:**
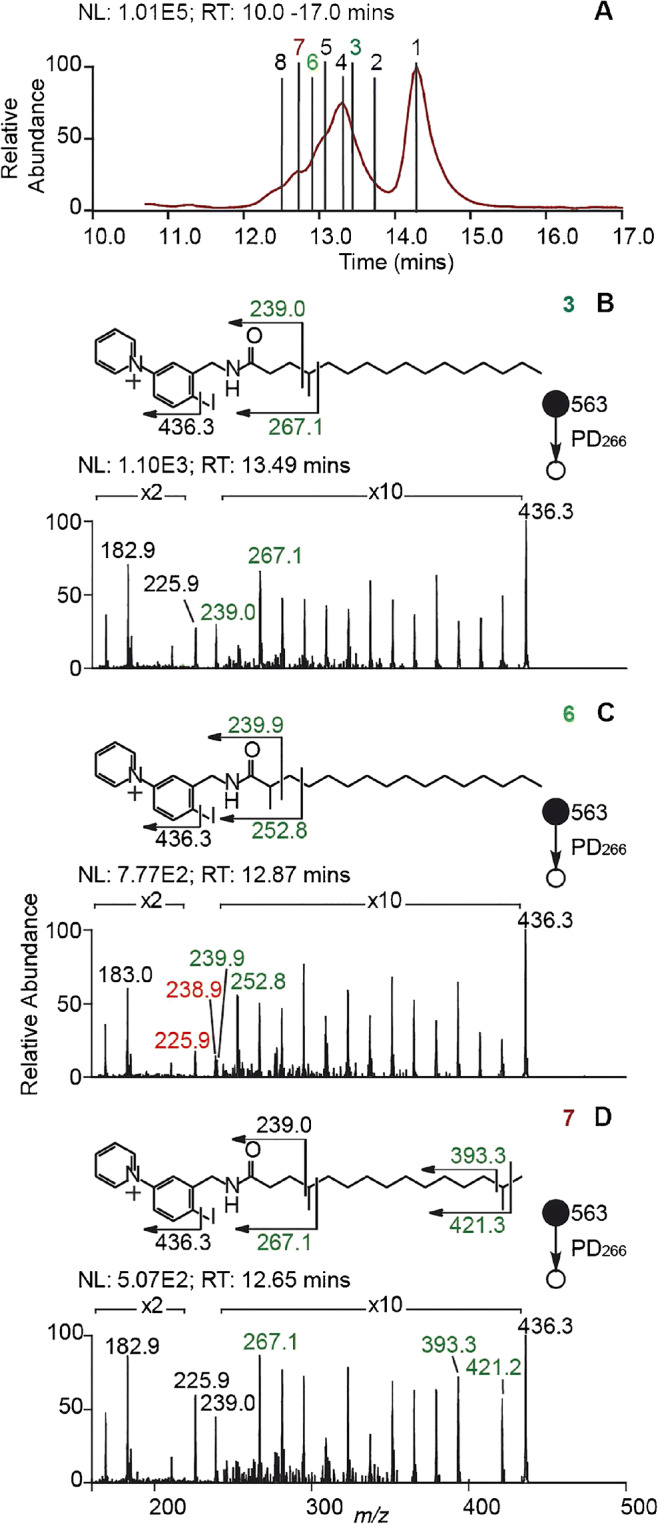
**A** Liquid chromatographic trace and associated **B**–**D** RDD tandem mass spectra of isomeric functionalized FAs extracted from *vernix caseosa*. Due to extensive FA fragmentation, methyl-branching isomers are distinguished (green signals) on the chromatographic time scale. Reprinted with permission from [84], copyright 2019 American Chemical Society

Despite these challenges, many research groups have demonstrated first promising results of advanced tandem MS methods applied during LC-MS^n^ experiments. In particular, Ducati et al. recently reported a new triple quadruple mass spectrometer with a modular fragmentation region for CID and EID experiments [47]. The authors succeeded to separate components of a mock mixture containing 114 metabolites via LC followed by CID-MS^2^ or EID-MS^2^. Although the former tandem MS method yielded highly abundant fragment ions consistent with CID databases, the use of the latter method improved structure identification due to EI-type fragment ions despite the low fragmentation efficiency. Light-based activation methods are readily combined with LC runs as long as the laser repetition rate or laser energy per pulse allow ion activation on chromatographic time scales. Two impressive examples of UVPD and RDD combined with chromatographic separation requiring only a single laser pulse were reported by Williams et al. [143] and Narreddula et al. [84] Using 193 nm UVPD, Brodbelt and co-workers showed that HCD of sodiated GPs followed by UVPD allows to obtain diagnostic fragment ions for DBs and *sn*-isomers [143]. For a mixture of PEs, this LC-HCD/UVPD-MS^3^ method helped to distinguish *sn*-isomers based on diagnostic product ions despite incomplete chromatographic separation. To combine improved ion detection with RDD, Narreddula et al. designed and synthesized a tailor-made compound that efficiently converts FAs into amides, contains a fixed charge, and homolytically loses iodine upon 266 nm laser irradiation [84]. This enables the detection of FAs after derivatization in positive ion mode and structural identification by RDD. As shown in Fig. 9, LC-MS of FAs from *vernix caseosa* results in a chromatographic trace with two broad features around 13.2 min and 14.2 min (Fig. 9A). RDD of these derivatized FAs results in extensive fragmentation. Three representative examples of isomeric species are shown in Fig. 9B–D. Due to radical-directed cleavage of virtually all carbon-carbon bonds, diagnostic fragment ion *m*/*z* values that indicate methyl-branching positions are obtained. These diagnostic fragment ions allow to assign individual FA species to sections of the broad chromatographic signal at 13.2 min.

Ion/molecule reactions have also been combined with LC-MS workflows [100, 144]. In particular, Kong et al. developed a pulsed-valve inlet system to consecutively react analytes with up to nine reagent gases on the chromatographic time scale [100]. By increasing the ozone partial pressure in the ozone–analyte interaction region compared to older setups to boost the reaction speed, Poad et al. succeeded in performing OzID of chromatographically separated GPs discerning DB isomers [144]. Although modifications of mass spectrometric equipment was necessary to combine ion/molecule reactions with LC-MS, optimization of the solution-phase composition and a new reaction chamber was required to adapt PB reactions for LC-MS^n^. Xia and co-workers used these optimized bioanalytic workflows to perform reversed-phase and hydrophilic interaction chromatography followed by PB-MS^2^ to identify head groups, FA chain lengths, and DB positions for a multitude of GPs in tissue, human plasma, and cancer cell extracts [128, 145]. The time requirements to collect action spectroscopic data is one of the major factors that hinder coupling of these MS-based spectroscopic tools with LC-MS routines. To overcome this obstacle, Oomens and co-workers used LC-MS for compound separation and automated sample fractionation followed by IRMPD spectroscopy of fractions of interest [146]. With this procedure, they were able to distinguish hydroxy-atorvastatin isomers formed upon enzymatic degradation of atorvastatin based on IRMPD spectra. Instead of sample fractionation, Schindler et al. reduced the flow of the LC system at elution times of preselected LC features achieving IRMPD spectroscopy differentiation of glycan isomers requiring only ~ 6 min per IRMPD spectrum instead of ~ 30 min [147].

## Mass spectrometry imaging and structure annotations

Another set of tools for MS-based metabolomics and lipidomics that have received considerable attention recently is MSI. MSI can disentangle the spatial heterogeneity of metabolite and lipid distributions within a sample unavailable from other MS measurements. The additional level of insight offered by MSI comes with a cost. The vast majority of MSI methods do not chromatographically separate ions prior to ion injection. This can lead to ion suppression or matrix effects and RTs are not available. MSI annotations are, thus, mostly based on the comparison of accurate mass measurements with available data basis. Therefore, on-tissue MS^n^I is pivotal for structure confirmation or annotation and revealing distributions of otherwise coalescing isobars or isomers. The major obstacle for on-tissue MS^n^I measurements is the limited sample material probed during a single MSI event complicating spatially and structurally resolved MSI experiments.

**Fig. 10:**
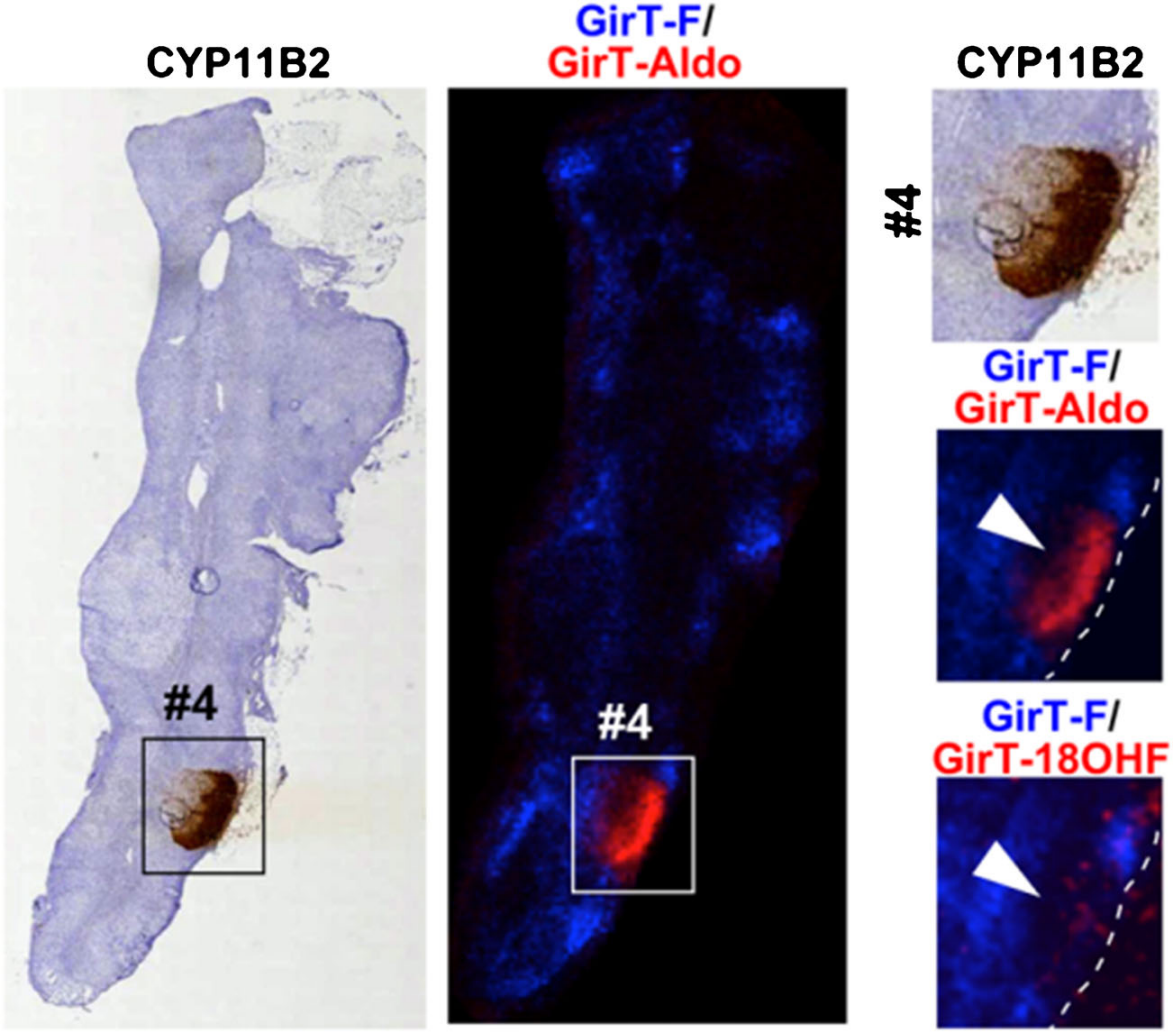
(Left) Stained human adrenal gland revealing aldo-producing cell clusters in brown. (Middle) MALDI-MS^3^I of GirT-derivatized steroids with 120 μm step size. (Right) Zoom-in of microcopy and MSI results. Adapted with permission from [149], copyright 2019 American Chemical Society

For this reason, specialized mass spectrometric equipment and optimized MSI workflows have been developed in order to spatially track specific compounds in tissues [148]. For example, Takeo et al. developed a method to track and distinguish steroids that typically show low MSI signal intensities and only differ by the position of hydroxy/carbonyl groups/DBs [149]. To increase mass spectrometric steroid signals and distinguish isomers, the authors derivatized steroids on-tissue with the Girard’s T (GirT) reagent carefully controlling the conditions to prevent analyte migration. The GirT-derivatized steroids exhibit increased mass spectrometric signals compared to experiments without GirT treatment due to introduction of a permanent charge. Additionally, GirT influences the fragmentation pathway of steroids in CID-MS^3^ experiments allowing isomer differentiation as highlighted in Fig. 10. Diagnostic fragments for the isomers glucocorticoid cortisol (F), 18-hydroxycortisol (18-OHF), and aldosterone (Aldo) after derivatization imaged with MALDI-MS^3^I show distinct lateral distributions in human adrenal gland. These distributions, especially for GirT-Aldo, are in line with results from histochemistry that probe the aldosterone synthase (CYP11B2). High abundance of GirT-Aldo is consistent with CYP11B2 positive regions, whereas other isomeric steroids are depleted in the same region. Drawbacks of this MS^3^I method are the sample preparation involving multiple error-prone steps and the moderate lateral resolution due to multiple fragmentation steps.

**Fig. 11:**
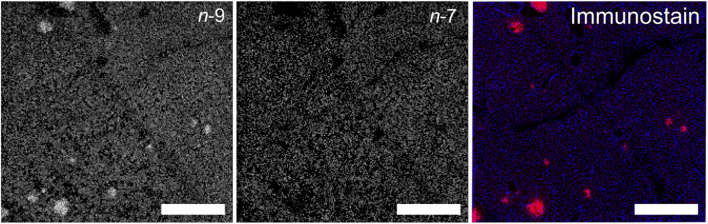
Reactive MALDI-MS^*2*^I of protonated PB-derivatized PC 34:1 from mouse pancreas. (Left, middle) MS images of *n*-9 and *n*-7 with a pixel resolution of 10 μm. (Right) Immunofluerescence after MSI experiments revealing β-cells in red and cell nuclei in blue. Scale bars are 600 μm. Adapted with permission from [152], copyright 2020 American Chemical Society

On-tissue derivatization cannot only help to identify steroid isomers in MS^n^I experiments but also facilitates identification of neurotransmitters as recently demonstrated by Andrén and co-workers [150]. The authors developed a compound that serves as MALDI matrix but also reacts with most neurotransmitters installing a permanent charge and enabling compound identification via MS^n^. The concept of reactive MALDI matrices for structure diagnostic MSI investigations has further been developed by Heiles and co-workers [151, 152]. Instead of reacting compounds with the matrix on-tissue, the authors identified benzophenone-based compounds that function as MALDI matrices but react on-demand, during UV laser irradiation, with DBs of lipids in a PB reaction. The resulting oxetanes allow to spatially discern DB position isomers as shown in Fig. 11 [152]. Reactive MALDI-MS^2^I of protonated PC 34:1 from mouse pancreas tissue with 10 μm lateral resolution reveals the presence of two distinct DB position isomers. Although the MS image for the signal assigned to a *n*-9 isomer contains circular regions with increased ion intensity, the corresponding *n*-7 isomers are downregulated in the same regions. Comparison to immunofluorescence images that reveal the location of β-cells (red) and all cell nuclei (blue) indicate that the regions of increased *n*-9 isomer abundances are in line with islets of Langerhans indicating underlying unknown biochemical events that lead to this local isomer enrichment.

But DB position isomers of lipids have also been assigned and localized with a number of other structure diagnostic MSI methodologies. For example, Brodbelt and co-workers developed a DESI-UVPD-MS^2^I method to track DB position isomers of FAs and GPs [153, 154]. Bednařík et al. used on-tissue PB derivatization followed by MALDI-MS^2^I with a specialized post-ionization setup to distinguish PC/PS DB positions in rat brain [155]. Also on-tissue epoxidation [156], on-tissue ozonolysis [126], OzID [157], ion/ion reactions [158], and EID [159] have been utilized to reveal different lipid isomers and track their distributions in tissues. Most of these reports demonstrate the performance characteristics of the developed bioanalytic tools but Young et al. used MALDI-OzID-MSI to show that the metabolic demand of cancer infection can alter canonical FA and GP formation, thereby creating lipids with unusual DB positions [160]. These lipid isomers and their association with breast cancer phenotypes suggest a functional role of these unsaturated lipids and showcase the power of advanced tandem MS methodologies in combination with MSI.

## Future perspectives and conclusion

In this review, the author set out to outline benefits and drawbacks of newly developed advanced tandem MS methods with the intention to offer insights into basic principles, progress in the field, and first applications. In contrast to high-throughput applications in MS-based untargeted metabolomics and lipidomics designed for large sample cohorts, most advanced tandem MS tools are often used in specialized studies by experts in the field rather than by a large number of users. One reason for this development is related to the limited access of non-experts to many of the herein described activation methods. But gradually more of these tandem MS methods will become commercially available as documented by EID and UVPD modules installed on SCIEX and Thermo Fisher Scientific mass spectrometers or multi-activation modalities such as EID, ECD, and UVPD as provided by the omnitrap by Fasmatech.

Another reason for the limited use of advanced tandem MS tools is the comparison of analytic figures of merit of CID-based untargeted workflows with other tandem MS strategies. Undoubtedly, CID is and will remain the workhorse in most studies that aim to assign metabolite structures and has been improved and optimized over generations of mass spectrometrists. On the other hand, CID, even when combined with LC or IMS separation, will most likely not allow to assign structures of unknown/unexpected metabolites on the isomer level for years to come. Therefore, a combination of CID with specialized tandem MS methodologies, even when they have inferior analytic performance compared to CID at the moment, will broaden the spectrum of structures available from untargeted metabolomic and lipidomic studies. Furthermore, developments of new and some established advanced tandem MS tools or corresponding mass spectrometers will most likely decrease the gap in performance between CID and other activation strategies.

Another aspect that hinders progress and broader appreciation of advanced tandem MS tools is the absence of consistent fragmentation rules and databases. Although numerous research groups have built extensive experimental or in silico CID databases for metabolites and currently explore the combination of the available data with deep learning tools or neuronal networks for tandem MS predictions, limited data is available for the methods discussed above. In order to progress with advanced tandem MS methodologies and make them available for a broader community, collaborations between mass spectrometry method developers, research groups employing standardized metabolomics/lipidomics, and bioinformaticians are urgently needed to establish UVPD, OzID, PB, spectroscopic approaches, or other tools as valuable addition to the method portfolio in untargeted metabolomic and lipidomic research.
